# Multi‐Target‐Directed Ligands (MTDLs) as Potential Therapeutic Candidates Targeting Multiple Pathogenic Factor of Alzheimer's Disease

**DOI:** 10.1002/cns.70919

**Published:** 2026-05-18

**Authors:** Jaewoon Jung, Yebeen Kim, Asifiwe Clarisse Cirunduzi, Seonghyun Yoon, Seokyoung Bang, Seung‐Hoon Yang

**Affiliations:** ^1^ Department of Biomedical Engineering, College of Life Science and Biotechnology Dongguk University Seoul Republic of Korea

**Keywords:** Alzheimer's disease (AD), amyloid‐β (aβ), multi‐target‐directed ligands (MTDLs), neuroinflammation, tau

## Abstract

**Background:**

Alzheimer's disease (AD) is the most common cause of dementia and a chronic neurodegenerative disorder in older adults. AD is not driven by a single factor but by the interaction of multiple pathological processes, including amyloid‐β (Aβ) accumulation, tau hyperphosphorylation, and chronic neuroinflammation. Aβ aggregates into plaques that disrupt neuronal signaling, while hyperphosphorylated tau forms neurofibrillary tangles, leading to neuronal loss. These processes act synergistically to amplify toxicity. Persistent activation of microglia and astrocytes further promotes neuroinflammation, worsening Aβ and tau pathology.

**Current Limitations:**

Single‐target therapies directed at Aβ or tau have shown limited clinical success and failed to alter disease progression, underscoring the complexity of AD.

**Objectives:**

In response, multi‐target‐directed ligands (MTDLs) have emerged as a promising strategy. By simultaneously modulating several disease pathways, MTDLs can inhibit Aβ aggregation, reduce tau phosphorylation, and exert antioxidant and anti‐inflammatory effects. This review summarizes recent progress on MTDLs, highlighting their mechanisms of action, representative drug candidates, and outcomes from preclinical and clinical studies.

**Conclusions:**

Multi‐target strategies have the potential to achieve more effective and disease‐modifying outcomes than conventional approaches. A critical evaluation of their opportunities and challenges may guide future therapeutic development and the advancement of precision medicine for Alzheimer's disease.

## Graphical abstract

Multi‐target‐directed ligands (MTDLs) offer a promising strategy for Alzheimer's disease by simultaneously modulating amyloid‐β, tau pathology, neuroinflammation, and oxidative stress. This review highlights representative MTDL candidates and their therapeutic potential in addressing the multifactorial nature of AD.

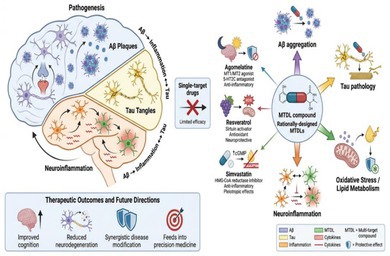

## 1 Introduction

Alzheimer's disease (AD) is a leading neurodegenerative disease affecting millions of people worldwide [1, 2], causing cognitive decline, memory loss, and behavioral changes [3, 4]. Despite significant advances in our understanding of the disease's molecular biology, effective fundamental treatments remain limited [5, 6]. Existing therapeutic approaches have primarily focused on single‐target strategies targeting a single pathological target (Aβ or tau protein), with limited success in clinical trials [7, 8]. This highlights the need for more complex and comprehensive treatment approaches.

While Aβ, tau, and neuroinflammation have been widely recognized as central features of AD, emerging evidence highlights the critical contribution of metabolic and lipid regulatory pathways in modulating disease progression. Lipid metabolism plays a central role in maintaining neuronal membrane integrity, regulating amyloid precursor protein (APP) processing, and shaping neuroinflammatory responses. Dysregulation of lipid homeostasis has been associated with altered amyloid‐β aggregation, tau pathology, and synaptic dysfunction, ultimately influencing cognitive outcomes.

Recent studies further support the functional relevance of lipid metabolism in neurological disorders. For instance, modulation of lipid metabolic pathways has been shown to significantly improve cognitive function following brain injury and ischemic events, underscoring its role in neuroprotection and recovery. Moreover, cerebrospinal fluid (CSF) multi‐omics analyses have identified lipid‐associated molecular signatures as key determinants of neurological outcomes, highlighting their importance in disease progression and patient stratification.

Collectively, these findings suggest that metabolic and lipid pathways are not merely secondary contributors but integral components of AD pathophysiology. Incorporating these axes into the conceptual framework of AD further strengthens the rationale for multi‐target‐directed ligands (MTDLs), which can simultaneously modulate central pathological processes and systemic metabolic dysfunction [9].

Aβ is a peptide produced by the enzymatic cleavage of APP (Amyloid precursor protein). When excessive accumulation occurs in the brain, it deposits in the form of insoluble plaques [10, 11]. These Aβ plaques induce inflammation around neurons, disrupting calcium homeostasis, impairing mitochondrial function, and damaging synapses [12, 13, 14]. While Aβ can be recognized and cleared by microglia, chronic activation can induce a persistent inflammatory response, further exacerbating neuronal damage [15, 16].

Tau is a protein that normally stabilizes microtubules, but in AD, it becomes hyperphosphorylated, leading to the formation of neurofibrillary tangles. Abnormal tau disrupts microtubule structure and interferes with intracellular transport, severely impairing neuronal function. Tau pathology is also exacerbated by an inflammatory environment, and studies have shown that inflammatory cytokines secreted by microglia, in particular, influence tau phosphorylation [17, 18, 19].

**FIGURE 1:**
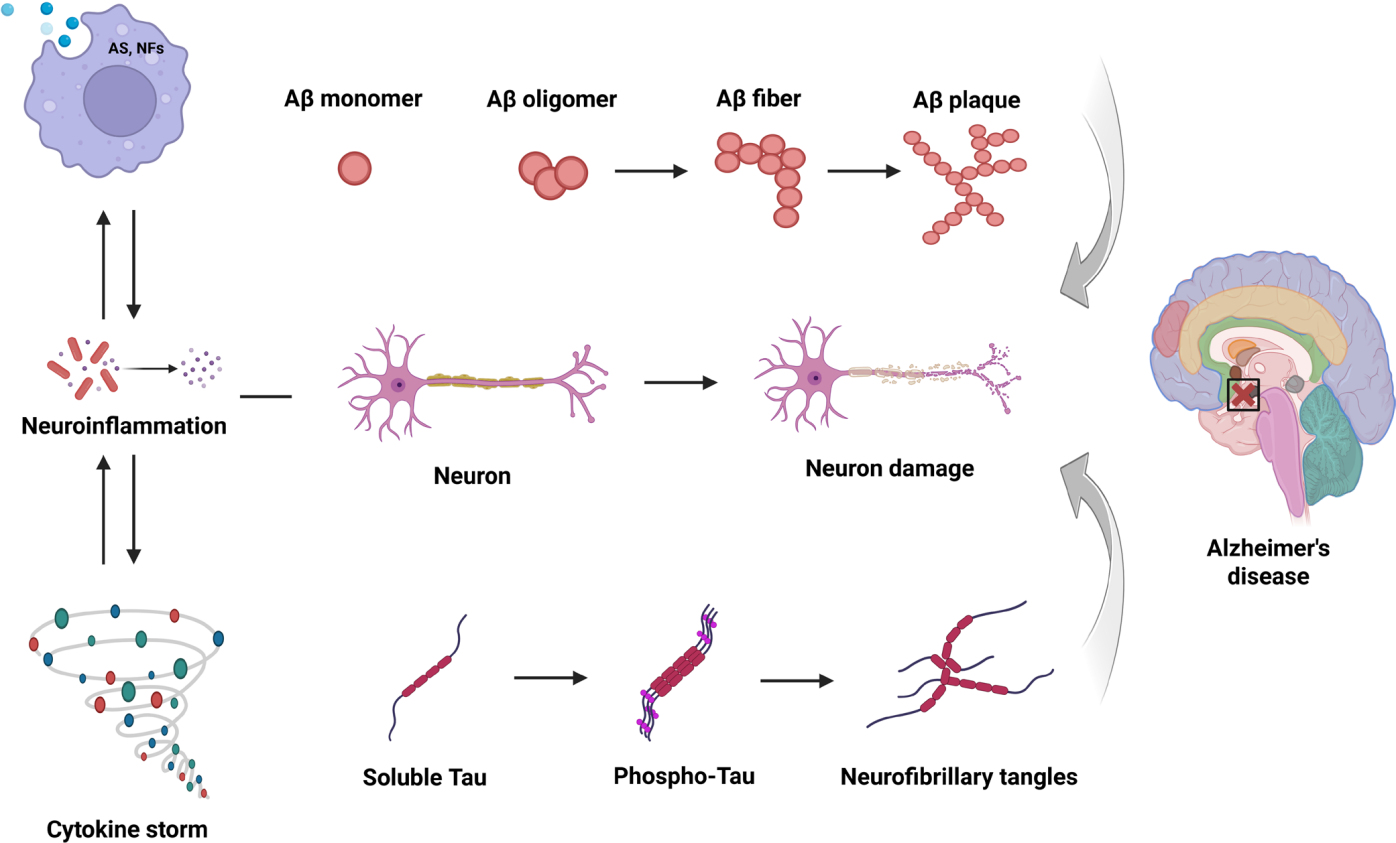
Interplay of microglial phenotypic shift, amyloid plaque deposition, and tau‐mediated neurofibrillary tangle formation in the progression of Alzheimer's disease. Schematic illustration of the interplay among microglia, astrocytes (AS), amyloid‐β (Aβ), tau, neurofilaments (NFs), and neurons. During the initial phase, microglia actively remove cellular debris and misfolded proteins through enhanced phagocytosis. Astrocytes support neuronal homeostasis by regulating synaptic transmission and maintaining extracellular ion balance. As disease progresses, microglia shift toward a pro‐inflammatory phenotype, releasing cytokines that amplify neuroinflammation, while reactive astrocytes further exacerbate inflammatory signaling and contribute to synaptic dysfunction. Concurrently, soluble Aβ gradually aggregates into extracellular plaques, whereas intracellular tau proteins undergo hyperphosphorylation and assemble into neurofibrillary tangles. These pathological hallmarks, together with axonal injury reflected by elevated NFs, further stimulate microglial and astrocytic activation, thereby reducing their protective phagocytic and homeostatic functions. This establishes a detrimental feedback loop that accelerates neuronal dysfunction and degeneration.

One of the pathological mechanisms that has recently attracted attention is neuroinflammation [20]. Neuroinflammation is primarily induced by the activation of microglia and astrocytes, which secrete inflammatory cytokines and chemokines, causing toxicity in surrounding neurons [21, 22, 23]. While neuroinflammation plays a crucial role in AD pathology, including recognition and removal of Aβ and the clearance of damaged cells [12, 24], chronic activation due to persistent stimulation can lead to the persistent secretion of inflammatory cytokines and the elimination of synapses, creating a vicious cycle that worsens neural network function and leads to neuronal cell death [25, 26]. Strategies to modulate this function and alleviate inflammatory conditions are considered important targets for AD treatment, as well as amyloid‐β and tau [27, 28](Figure 1).

To simultaneously target the diverse causes of AD, the development of multi‐targeting ligands (MTDLs) is actively being utilized [29, 30]. In this regard, it is important to clearly define multi‐target‐directed ligands (MTDLs) and distinguish them from repurposed or pleiotropic drugs. MTDLs are rationally designed single molecular entities that intentionally interact with two or more disease‐relevant targets with defined pharmacological activity. In contrast, repurposed drugs may exhibit multiple biological effects; however, these effects are often indirect or secondary consequences rather than the result of deliberate multi‐target optimization. Therefore, while both approaches may influence multiple pathological pathways, MTDLs are characterized by intentional design and balanced activity across targets, which is particularly important for addressing complex diseases such as Alzheimer's disease. Based on this definition, MTDLs are expected to exert coordinated pharmacological effects across multiple pathological pathways. These compounds possess diverse pharmacological properties, including inhibition of Aβ accumulation [31], regulation of tau protein, and antioxidant and anti‐inflammatory effects, allowing them to act multi‐facetedly on the pathogenesis of AD [32, 33]. Because AD pathogenesis is driven by the interaction of multiple pathological factors rather than a single etiology [34, 35], single‐targeting strategies inevitably have limitations in disease suppression [36]. MTDLs, which simultaneously modulate various factors, including neuroinflammation, Aβ, and tau pathology, are emerging as a new therapeutic approach for AD. This review examines various MTDLs as a novel therapeutic approach to more effectively address the complexity of AD. Beyond their pharmacological characterization, recent computational and systems biology approaches further support the rationale for multi‐target strategies in Alzheimer's disease. AD is increasingly recognized as a network‐level disorder involving interconnected molecular and cellular pathways rather than isolated pathological events. In this context, multiplex disease network models and dual‐ranking algorithms have been developed to identify key disease‐associated nodes and their interactions within complex biological systems [37]. These approaches highlight that modulating multiple interconnected targets may be more effective than single‐node interventions, thereby aligning with the conceptual framework of multi‐target‐directed ligands (MTDLs). Taken together, these perspectives further reinforce the need for integrative and multi‐target therapeutic strategies in Alzheimer's disease.

We explore the underlying mechanisms of neuroinflammation in AD, highlight key compounds with multi‐targeting activity, and discuss their therapeutic potential based on preclinical and clinical studies. We explore the underlying mechanisms of neuroinflammation in AD, highlight key compounds with multi‐targeting activity, and discuss their therapeutic potential based on preclinical and clinical studies.

## 2 Integrated Pathophysiology of Alzheimer's Disease: Linking Amyloid‐β, Tau, and Neuroinflammation

### 2.1 Amyloid‐β and Tau Proteins

According to the conventional amyloid cascade hypothesis, amyloid‐β (Aβ) accumulation is the initial cause of Alzheimer's disease [38, 39], followed by a cascade of pathological processes, including tau protein modification, neuroinflammation, and neuronal cell death [40]. However, recent research has highlighted the interplay between Aβ and tau proteins, exacerbating neurodegeneration [41, 42]. Understanding their complex pathological mechanisms is emerging as a critical challenge in developing treatment strategies. Aβ is produced by the cleavage of amyloid precursor protein (APP) [43]. While it is normally cleared under physiological conditions, in Alzheimer's disease it accumulates in the brain, forming plaques (amyloid plaques) [44, 45]. This disrupts signaling between neurons, activates microglia, induces chronic neuroinflammation, and promotes neuronal death [26, 46, 47].

Meanwhile, tau protein normally stabilizes microtubules within neurons, but in Alzheimer's disease, it becomes hyperphosphorylated and abnormally aggregates, forming neurofibrillary tangles (NFTs) [48]. This process leads to impaired transport of substances within neurons, impaired mitochondrial function, and dysfunction of the protein degradation system, ultimately leading to neuronal degeneration and death [49, 50]. In the pathological process of Alzheimer's disease, Aβ and tau proteins are reported to interact with each other to amplify neurodegeneration rather than acting individually [51, 52]. Aβ induces tauopathy by activating signaling pathways that promote hyperphosphorylation of tau protein [53]. In particular, activation of kinases such as GSK‐3β (Glycogen synthase kinase‐3β) increases abnormal phosphorylation of tau protein, leading to the formation of neurofibrillary tangles [50, 54, 55].

As tau protein alteration progresses, the toxicity of Aβ becomes more pronounced and neuronal cell death is promoted. This suggests that Aβ accumulation induces tau protein alteration, and the altered tau, in turn, increases Aβ neurotoxicity, forming a feedback loop [12, 53, 56]. Because existing hypotheses have difficulty fully explaining the pathological characteristics of Alzheimer's disease [57, 58], recent studies are focusing on elucidating the relationship between tau protein alterations and Aβ. Therefore, when establishing a treatment strategy for Alzheimer's disease, an approach that simultaneously targets Aβ and tau proteins is necessary, and clearly elucidating the pathological linkage mechanism between the two proteins will be an important direction for future research.

### 2.2 Neuroinflammation

Neuroinflammation is a core pathological mechanism of Alzheimer's disease [59], and it has recently been recognized as a leading factor that directly influences the initiation and progression of the disease [26], rather than a simple secondary reaction. Neuroinflammation is induced by the activation of microglia and astrocytes [25], which secrete various inflammatory mediators in response to pathological stimuli [60]. While initially, this response may help clear amyloid‐β (Aβ), clear damaged cells, and maintain homeostasis, a persistent and chronic inflammatory environment can have detrimental consequences [61]. Chronically activated microglia secrete excessive amounts of inflammatory cytokines, such as TNF‐α, IL‐1β, IL‐6, and MCP‐1 [62], which induce neuronal apoptosis, impair synaptic function, and negatively impact neural circuits in the hippocampus and prefrontal cortex [63, 64]. This inflammatory response increases the permeability of the blood–brain barrier (BBB) [65, 66], inducing the infiltration of peripheral immune cells into the brain and further exacerbating the inflammatory response within the brain [67]. Furthermore, neuroinflammation induces oxidative stress in neurons, leading to the accumulation of amyloid‐β and the hyperphosphorylation and aggregation of tau proteins [68, 69, 70]. These pathological changes ultimately lead to key clinical symptoms of Alzheimer's disease, such as memory decline and cognitive dysfunction [12, 71, 72]. Meanwhile, recent studies have reported that microglia, among those responsible for the inflammatory response, are selectively activated during the progression of AD and play a crucial role in regulating inflammatory responses as well as in clearing Aβ [16, 24, 73]. This suggests that strategies that target specific phenotypes or reprogramming microglia, rather than simply suppressing their function, are therapeutically important [74]. Importantly, recent studies suggest that microglial activation is not solely governed by classical inflammatory signaling but is also shaped by cellular metabolic states and nutrient availability. Glial cells, including microglia and astrocytes, function as metabolic sensors that integrate signals from glucose, lipid, and amino acid metabolism, thereby regulating their activation states and inflammatory profiles. Disruptions in these nutrient‐sensing pathways can contribute to maladaptive glial responses and sustained neuroinflammation [75].

Consistent with this, accumulating experimental evidence indicates that direct modulation of neuroinflammatory pathways can lead to functional recovery of cognitive deficits. For example, apelin‐13, an endogenous peptide ligand of the APJ receptor, has been shown to alleviate neuroinflammation and improve memory impairment in AD‐like models. Mechanistically, apelin‐13 reduces microglial activation and pro‐inflammatory cytokine production while enhancing neuroprotective signaling through the BDNF–TrkB pathway, which is critically involved in synaptic plasticity and memory formation. In vivo studies further demonstrated that apelin‐13 administration attenuates neuroinflammatory responses and rescues learning and memory deficits, providing direct evidence that targeting neuroinflammation can translate into cognitive benefits [76].

In addition to central nervous system–intrinsic mechanisms, emerging evidence suggests that peripheral factors, including gut–brain interactions, may also contribute to Alzheimer's disease pathophysiology. The gut microbiota and their metabolites have been implicated in modulating neuroinflammation, amyloid‐β deposition, and cognitive function in AD‐like models. Furthermore, microbiota‐targeted approaches are being explored as potential adjunct strategies for AD treatment [77, 78]. In addition to these regulatory mechanisms, emerging evidence suggests that cellular senescence represents another important contributor to Alzheimer's disease pathophysiology, closely interacting with oxidative stress and neuroinflammation. Senescent‐like phenotypes have been identified in neurons and glial cells, where they contribute to chronic inflammatory signaling and tissue dysfunction. Notably, recent studies have demonstrated that compounds such as icariin can modulate neuronal senescence by targeting p53‐dependent pathways, thereby alleviating AD‐related pathology and cognitive impairment in experimental models [79]. These findings further support the notion that senescence‐related pathways may serve as an additional therapeutic axis. Together, these findings indicate that neuroinflammation is a dynamic and modifiable process shaped by both intrinsic and extrinsic factors, reinforcing its role as a key therapeutic target and supporting the rationale for multi‐target strategies in Alzheimer's disease. Therefore, neuroinflammation closely interacts with Aβ accumulation, tau pathology, synaptic damage, and BBB disruption, forming a complex network among the pathophysiological mechanisms of Alzheimer's disease [80, 81]. The interplay between these pathological mechanisms forms a feedback loop, further accelerating disease progression [82]. Therefore, it is crucial to recognize neuroinflammation as a factor that must be integratively controlled alongside Aβ and tau pathology, rather than viewing it as a single therapeutic target. Considering this complex pathophysiology, the therapeutic limitations of current single‐target drugs are clear, and the development of multitarget therapies (multitarget‐directed ligands, MTDLs) capable of simultaneously modulating multiple pathological factors is gaining traction [83, 84].

## 3 Representative Multi‐Target Agents in Current Alzheimer's Research

Multi‐target therapeutics have the potential to simultaneously modulate the complex pathophysiology of Alzheimer's disease (Aβ accumulation, tau pathology, neuroinflammation, etc.). The following substances possess diverse physiological activities, making them suitable for multi‐targeting. They are also highly safe for long‐term use and exhibit excellent anti‐inflammatory and antioxidant effects. Furthermore, they offer greater structural diversity and optimization potential than existing drugs, and their potential for preventive treatment has led to their recognition as highly promising resources for AD treatment strategies, and extensive research is underway.

### 3.1 Agomelatine

While the direct binding or metabolism modulation of Aβ has not been clearly elucidated, Agomelatine may influence amyloid‐β pathology through indirect mechanisms [85]. Specifically, it has been reported to reduce Aβ accumulation and toxicity by suppressing oxidative stress and improving the brain environment. The associated increase in antioxidant enzyme activity and reduction in ROS may contribute to mitigating Aβ‐induced cellular damage [86].

While Agomelatine's direct action on tau protein is unknown, it has been suggested that it may inhibit tau hyperphosphorylation through pathways associated with inflammation and oxidative stress mitigation [86, 87]. Since oxidative stress activates tau kinases such as GSK‐3β [88, 89], Agomelatine's antioxidant effects may serve as an indirect mechanism for regulating tau pathology [86].

Agomelatine suppresses the expression of inflammatory cytokines such as TNF‐α and IL‐6 [90], and inhibits NF‐κB signaling, a key regulator of inflammatory responses in the brain [91, 92]. It regulates microglial activation and reduces oxidative damage, contributing to an overall anti‐inflammatory environment. This is crucial for disrupting the chronic inflammatory loop in AD [86, 93]. Agomelatine is a drug that simultaneously exerts sleep regulation, mood stabilization, antioxidants, and anti‐inflammatory effects through melatonin receptor (MT1/MT2) agonism and serotonin receptor (5‐HT2C) antagonism [94, 95]. This complex pharmacological action is considered a potential candidate for multi‐targeting strategies, as it can simultaneously modulate multiple axes of AD's pathological mechanisms [96].

In cell experiments, Agomelatine significantly reduced levels of Aβ‐induced oxidative stress markers such as MDA, LDH, and ROS, which are interpreted as a result of the restoration of antioxidant enzyme systems. Simultaneously, it inhibited tau protein phosphorylation through modulation of the PTEN/Akt/GSK‐3β signaling pathway, significantly improving cell viability [86]. Studies using the APP mouse model have shown that Agomelatine reduces Aβ plaque accumulation, tau pathology, and neuroinflammation, and improves spatial memory [87]. Furthermore, it has been shown to alleviate oxidative stress and inflammation and restore synaptic plasticity in the hippocampus [97, 98]. Clinically, limited evidence suggests that agomelatine may improve certain behavioral and psychological symptoms in patients with AD, such as agitation and aggression [99]. Although these findings are based on small‐scale studies. Larger clinical trials are currently underway to further evaluate its potential.

Overall, while agomelatine exhibits multi‐target pharmacological properties and promising preclinical effects, its therapeutic efficacy in Alzheimer's disease has not yet been fully established. Therefore, it should be regarded as a potential repurposing candidate rather than a confirmed disease‐modifying agent, and further large‐scale, AD‐specific clinical studies are required to determine its efficacy and long‐term safety. Agomelatine exhibits a relatively well‐defined target profile through MT1/MT2 receptor agonism and 5‐HT2C antagonism, enabling simultaneous modulation of circadian rhythm and neuroinflammatory pathways. It demonstrates favorable blood–brain barrier (BBB) penetration, supporting central nervous system activity. However, its pharmacokinetic properties are characterized by low and variable oral bioavailability due to extensive first‐pass metabolism. While its safety profile is supported by established clinical use in depression, its translational application in Alzheimer's disease remains limited by the lack of robust AD‐specific clinical evidence and long‐term efficacy data.

### 3.2 Resveratrol

Resveratrol has been reported to alter the APP processing pathway, inhibiting Aβ production [100]. It also prevents the aggregation of pre‐formed Aβ and reduces its toxicity [101]. It also increases the expression of Aβ‐degrading enzymes (IDE and neprilysin), which are crucial for Aβ clearance [102]. Preclinical studies have shown that long‐term administration of resveratrol to an APP/PS1 mouse model reduces Aβ plaque burden and inhibits oligomeric Aβ accumulation [103, 104]. Specifically, resveratrol has been demonstrated in vivo to activate the SIRT1 pathway, inhibiting tau protein acetylation and reducing GSK‐3β activity, thereby inhibiting tau hyperphosphorylation and NFT formation. This effect is linked to the maintenance of intraneuronal transport systems and the prevention of axonal transport disorders, resulting in improved memory and cognitive function in behavioral experiments [105]. At the cellular level, analysis confirmed that Aβ‐induced cytotoxicity and ROS production were significantly reduced in SH‐SY5Y neurons after resveratrol treatment, attributing this to its potent antioxidant properties [106, 107].

The mechanism of inflammation regulation was also elucidated in preclinical studies [108]. In an experiment using LPS‐activated BV2 microglial cells, resveratrol significantly reduced the expression of inflammatory factors such as TNF‐α and IL‐1β by inhibiting nuclear translocation of NF‐κB [109, 110]. Furthermore, increased antioxidant enzyme expression was observed [111, 112]. Thus, resveratrol is a representative agent that comprehensively regulates various pathologies, including Aβ, tau, inflammation, and oxidative stress. Limitations of resveratrol include its low bioavailability and limited blood–brain barrier (BBB) penetration, which were observed in preclinical studies [113, 114]. To overcome these limitations, nanoparticle formulations, liposomes, and highly soluble prodrugs are being developed in parallel [115]. Indeed, studies have demonstrated in vivo that administration of resveratrol nanoparticle formulations increases brain drug concentration and enhances efficacy compared to conventional formulations [116, 117]. Recent pilot clinical trials have demonstrated a certain level of biomarker improvement and safety, and expansion into large‐scale clinical trials is warranted [118, 119].

Although resveratrol exhibits broad multi‐target pharmacological activities, its clinical applicability as a direct therapeutic agent for Alzheimer's disease remains constrained by its low bioavailability and limited blood–brain barrier penetration. Therefore, rather than being considered a fully viable MTDL in its native form, resveratrol may be more appropriately viewed as a structural and mechanistic template for the development of improved derivatives and delivery systems. In this context, strategies such as nanoformulations, prodrug design, and chemical modification are likely to play a critical role in translating its multi‐target potential into clinically relevant outcomes. Overall, resveratrol exhibits one of the broadest target profiles among MTDLs, simultaneously modulating amyloid‐β aggregation, tau pathology, oxidative stress, and neuroinflammatory signaling pathways. However, its therapeutic translation is fundamentally limited by poor bioavailability, rapid metabolism, and insufficient effective brain exposure. While it is generally well tolerated, these pharmacokinetic constraints emphasize the need for optimized formulations or derivatives to realize its clinical potential.

### 3.3 Simvastatin

Simvastatin inhibits cholesterol synthesis by inhibiting HMG‐CoA reductase, which alters the liposome environment of APP involved in Aβ production and reduces the accessibility of β‐secretase [120, 121]. Furthermore, increased Aβ‐degrading enzyme activity has been reported, and its overall effect on plaque accumulation is expected to be suppressed [122, 123].

Preclinical studies have shown that long‐term simvastatin administration in an APP/PS1 mouse model significantly reduced Aβ plaque accumulation in brain tissue, which was closely associated with decreased β‐secretase protein expression [124]. In particular, simvastatin has been demonstrated to alter the structure of cholesterol‐rich lipid rafts in the brain, thereby directing APP processing toward a non‐amyloidogenic pathway [125].

Tau phosphorylation is closely related to cholesterol metabolism and inflammatory responses [126, 127]. Simvastatin may inhibit NFT formation by regulating the expression of tau protein kinases (e.g., GSK‐3β) [128]. Cholesterol regulation also affects brain cell membrane stability and indirectly affects tau pathological accumulation [129, 130].

In cell experiments, simvastatin treatment significantly reduced Aβ oligomer‐induced neurotoxicity in SH‐SY5Y cells, suppressing ROS production and increasing cell viability [131, 132]. Simvastatin exhibits potent anti‐inflammatory effects by inhibiting the NF‐κB pathway, reducing inflammatory cytokine production, and improving vascular endothelial function [133, 134, 135]. Specifically, improved vascular function, along with increased cerebral blood flow, positively influences inflammation mitigation and neuronal protection [136].

Preclinical studies suggest that simvastatin has the potential to be a MTDL agent that comprehensively modulates key axes of Alzheimer's disease pathophysiology through multiple mechanisms: inhibition and promotion of Aβ production and clearance, suppression of tau pathology, modulation of inflammation and oxidative stress, and improvement of vascular function [137]. Currently, some epidemiological studies have shown a lower incidence of AD in statin‐treated patients, and clinical trials and meta‐analyses related to this topic are actively underway [138]. Although some clinical trials have observed positive trends in cognitive function maintenance, these are mainly small‐scale and short‐term studies, and large‐scale, long‐term clinical trials are required in the future.

Clinical findings regarding the cognitive benefits of simvastatin remain inconsistent, with several studies reporting limited or no significant improvement in cognitive outcomes. These mixed results suggest that, despite its promising multi‐target mechanisms observed in preclinical models, the translation of simvastatin's effects into meaningful clinical benefits remains uncertain. Factors such as patient heterogeneity, disease stage, treatment duration, and differences in study design may contribute to these discrepancies. Therefore, simvastatin should be interpreted as a potential multi‐target agent with context‐dependent efficacy, and further well‐designed clinical studies are required to clarify its therapeutic role in Alzheimer's disease. Overall, simvastatin exhibits a multi‐target profile primarily centered on cholesterol metabolism, with secondary effects on amyloid processing, tau phosphorylation, and neuroinflammatory pathways. Its lipophilic nature enables blood–brain barrier penetration, and its pharmacokinetic and safety profiles are well established through long‐term clinical use. However, inconsistent clinical outcomes in cognitive endpoints remain a major translational challenge, suggesting that its therapeutic efficacy may depend on disease stage, patient characteristics, and treatment duration.

### 3.4 Sildenafil

Sildenafil, originally developed as a treatment for erectile dysfunction, is a PDE5 inhibitor that increases cyclic GMP (cGMP) levels, leading to vasodilation and improved blood flow [139, 140]. In addition to improving cerebral blood flow, it also influences signaling pathways within the brain, suggesting its potential application in the management of AD pathology [141, 142].

Preclinical studies have reported that sildenafil administration suppresses Aβ production and promotes its degradation by modulating β/γ‐secretase activity [143, 144]. Furthermore, inhibition of GSK‐3β, which is mediated by cGMP‐PKG pathway activation, has been shown to reduce tau phosphorylation and NFT formation [145, 146]. Notably, long‐term sildenafil administration (4–8 weeks) in APP/PS1 and 3xTg‐AD mouse models reduced Aβ plaque burden and inhibited soluble Aβ oligomers, which also led to improved cognitive function [147, 148]. Furthermore, synaptic function and neuronal survival were promoted through the CREB‐BDNF‐NGF pathway, and behavioral memory enhancement was observed [143, 147, 148].

Even in an inflammatory environment, Sildenafil demonstrated a marked anti‐inflammatory effect [149]. Sildenafil treatment suppressed the expression of inflammatory cytokines such as TNF‐α and IL‐1β, and this was closely associated with inhibition of the NF‐κB signaling pathway [148, 150]. Based on these preclinical data, a large‐scale epidemiological analysis revealed a significant reduction in the risk of developing AD in patients taking PDE5 inhibitors [151]. A Phase 2 clinical trial evaluating the potential of Sildenafil as an AD treatment is currently underway, and preparations for Phase 3 clinical trials are also actively underway [152]. While these findings are encouraging, it is important to interpret epidemiological associations with caution, as observational data do not establish causal relationships. The reported reduction in AD risk among PDE5 inhibitor users may be influenced by confounding factors such as comorbidities, lifestyle, or healthcare access. Therefore, despite promising preclinical evidence and ongoing clinical trials, the efficacy and long‐term safety of Sildenafil for cognitive outcomes in Alzheimer's disease remain to be fully established. Robust evidence from well‐controlled, large‐scale clinical trials will be necessary to determine its true therapeutic potential. Sildenafil differs from other MTDLs in that its primary target profile is centered on PDE5 inhibition and subsequent activation of the cGMP–PKG signaling pathway, linking vascular, neurotrophic, and anti‐inflammatory mechanisms. It exhibits favorable blood–brain barrier accessibility and pharmacokinetic properties, including rapid absorption and predictable systemic exposure. Its safety profile is well established in approved indications such as erectile dysfunction and pulmonary hypertension. However, its translational potential in Alzheimer's disease remains uncertain, as current evidence is largely based on preclinical studies and observational data, and its long‐term efficacy and safety for cognitive outcomes require confirmation through well‐controlled clinical trials.

While all four agents exhibit multi‐target properties, their pharmacological profiles and translational potential differ substantially. Agomelatine and sildenafil demonstrate favorable blood–brain barrier (BBB) permeability and established safety profiles due to their prior clinical use, supporting their potential for rapid repurposing. In contrast, resveratrol exhibits one of the broadest target profiles but is significantly limited by poor bioavailability and insufficient brain exposure, which constrain its clinical applicability. Simvastatin, although supported by extensive clinical use and BBB penetration, has shown inconsistent outcomes in cognitive endpoints, highlighting challenges in translating multi‐target mechanisms into therapeutic efficacy (Table 1).

**TABLE 1:** Comparative overview of representative multi‐target‐directed ligands (MTDLs) in Alzheimer's disease.

Agent	Primary target profile	BBB permeability	Pharmacokinetic features	Development stage	Key limitations/Translational challenges	References
Agomelatine	MT1/MT2 agonist; 5‐HT2C antagonist; anti‐inflammatory and antioxidant effects	Good CNS penetration	Low and variable oral bioavailability; extensive hepatic metabolism	Clinically approved (depression); repurposing stage for AD	Limited AD‐specific clinical evidence; pharmacokinetic variability	[85, 86, 87, 88, 89, 90, 91, 92, 93, 94, 95, 96, 97, 98, 99]
Resveratrol	Aβ inhibition; tau modulation; antioxidant; anti‐inflammatory; SIRT1 activation	Limited effective brain exposure	Poor bioavailability; rapid metabolism	Preclinical to early clinical	Low bioavailability; requires formulation or derivative optimization	[100, 101, 102, 103, 104, 105, 106, 107, 108, 109, 110, 111, 112, 113, 114, 115, 116, 117, 118, 119]
Simvastatin	Cholesterol metabolism regulation; indirect Aβ modulation; anti‐inflammatory; vascular effects	BBB‐permeable (lipophilic statin)	Well‐characterized PK; CNS cholesterol modulation	Clinically approved (cardiovascular); investigated for AD	Inconsistent cognitive outcomes; patient heterogeneity	[120, 121, 122, 123, 124, 125, 126, 127, 128, 129, 130, 131, 132, 133, 134, 135, 136, 137, 138]
Sildenafil	PDE5 inhibition; cGMP–PKG signaling; vascular, neurotrophic, anti‐inflammatory effects	CNS‐accessible	Rapid absorption; relatively short half‐life	Approved (ED, PAH); Phase 2/3 trials for AD	Lack of causal clinical evidence; long‐term cognitive efficacy unclear	[139, 140, 141, 142, 143, 144, 145, 146, 147, 148, 149, 150, 151, 152]

These differences underscore that, beyond target multiplicity, factors such as pharmacokinetics, BBB permeability, and clinical evidence critically determine the translational success of multi‐target agents in Alzheimer's disease.

## 4 Future Perspectives and Challenges in MTDL Development

These compounds each demonstrate potential as multi‐target drugs (MTDLs) capable of simultaneously modulating various pathological axes of Alzheimer's disease, including inhibition of Aβ production, regulation of tau pathology, mitigation of neuroinflammation, inhibition of oxidative stress, and improvement of cerebral blood flow [83, 153, 154]. Agomelatine and sildenafil are already used for various indications and have established safety and BBB permeability, offering the advantage of rapid repurposing [96, 155]. In addition to the representative compounds discussed above, a growing number of rationally designed multi‐target‐directed ligands (MTDLs) have been actively investigated in both preclinical and clinical settings. These include hybrid molecules that combine acetylcholinesterase (AChE) inhibition with antioxidant or anti‐amyloid functionalities, as well as dual inhibitors targeting both cholinergic signaling and monoamine oxidase (MAO) activity.

Importantly, unlike repurposed agents, these compounds are deliberately designed to simultaneously engage multiple disease‐relevant targets with balanced pharmacological activity, thereby fulfilling the defining criteria of MTDLs. In addition, metal‐chelating MTDLs capable of modulating metal ion dyshomeostasis alongside anti‐inflammatory and antioxidative mechanisms have also been widely explored.

Some representative classes, including donepezil‐ and tacrine‐derived hybrids, have demonstrated promising neuroprotective effects in experimental models and have progressed into early‐phase clinical evaluation, highlighting the translational potential of medicinal chemistry–driven multi‐target strategies in Alzheimer's disease. Future research should explore strategies such as pinpointing pathological mechanisms through single‐cell omics and spatial transcriptome analysis, enhancing BBB permeability using delivery technology, elucidating mechanisms of action at the circuit level, and designing clinical trials. Furthermore, real‐time monitoring of drug response and advancement of treatment response prediction algorithms are expected to determine the ultimate success of AD treatment development. Recent advances in digital health technologies further support this perspective. Digital biomarkers derived from wearable devices, cognitive assessments, and neuroimaging data, combined with artificial intelligence (AI)‐based predictive models, have been increasingly applied to monitor disease progression and treatment response in Alzheimer's disease [156]. These approaches enable continuous, real‐time assessment of patient‐specific trajectories and may facilitate the dynamic adjustment of multi‐target therapeutic regimens within a precision medicine framework.

Building on these developments, recent studies provide concrete examples of how such approaches can directly inform MTDL design. For instance, the integration of network pharmacology with transcriptomic analysis has been used to identify multi‐target mechanisms, including coordinated anti‐apoptotic pathways, highlighting how multiple targets can be rationally selected based on system‐level data [157]. In addition, multimodal imaging approaches, such as diffusion tensor imaging analysis along the perivascular space (DTI‐ALPS) combined with hippocampal microstructural mapping, have been applied to delineate stage‐specific pathological alterations in Alzheimer's disease [158]. These strategies provide a framework for tailoring target combinations according to disease stage and individual patient characteristics, thereby enabling more precise and effective multi‐target drug design.

Despite these promising multi‐target characteristics, an important limitation remains in how the combined effects of these agents are currently evaluated. Although the above agents exhibit multi‐target activities across diverse pathological pathways, their effects are primarily described in a qualitative manner, and it remains unclear whether these actions produce additive or truly synergistic therapeutic outcomes. In the context of Alzheimer's disease, where multiple interconnected mechanisms drive disease progression, distinguishing between independent and synergistic effects is essential for accurately evaluating multi‐target strategies. Recent studies have suggested that coupled pharmacokinetic–pharmacodynamic (PK–PD) modeling can be used to quantitatively characterize such interactions. These approaches enable the integration of multiple drug actions and allow for the identification of synergistic effects by analyzing how combined mechanisms influence overall therapeutic outcomes [159].

Therefore, applying quantitative frameworks to these multi‐target agents will be important for clarifying whether their observed effects reflect true pharmacological synergy or simply parallel actions, ultimately improving the rational design and evaluation of MTDLs.

## 5 Discussion and Conclusion

**FIGURE 2:**
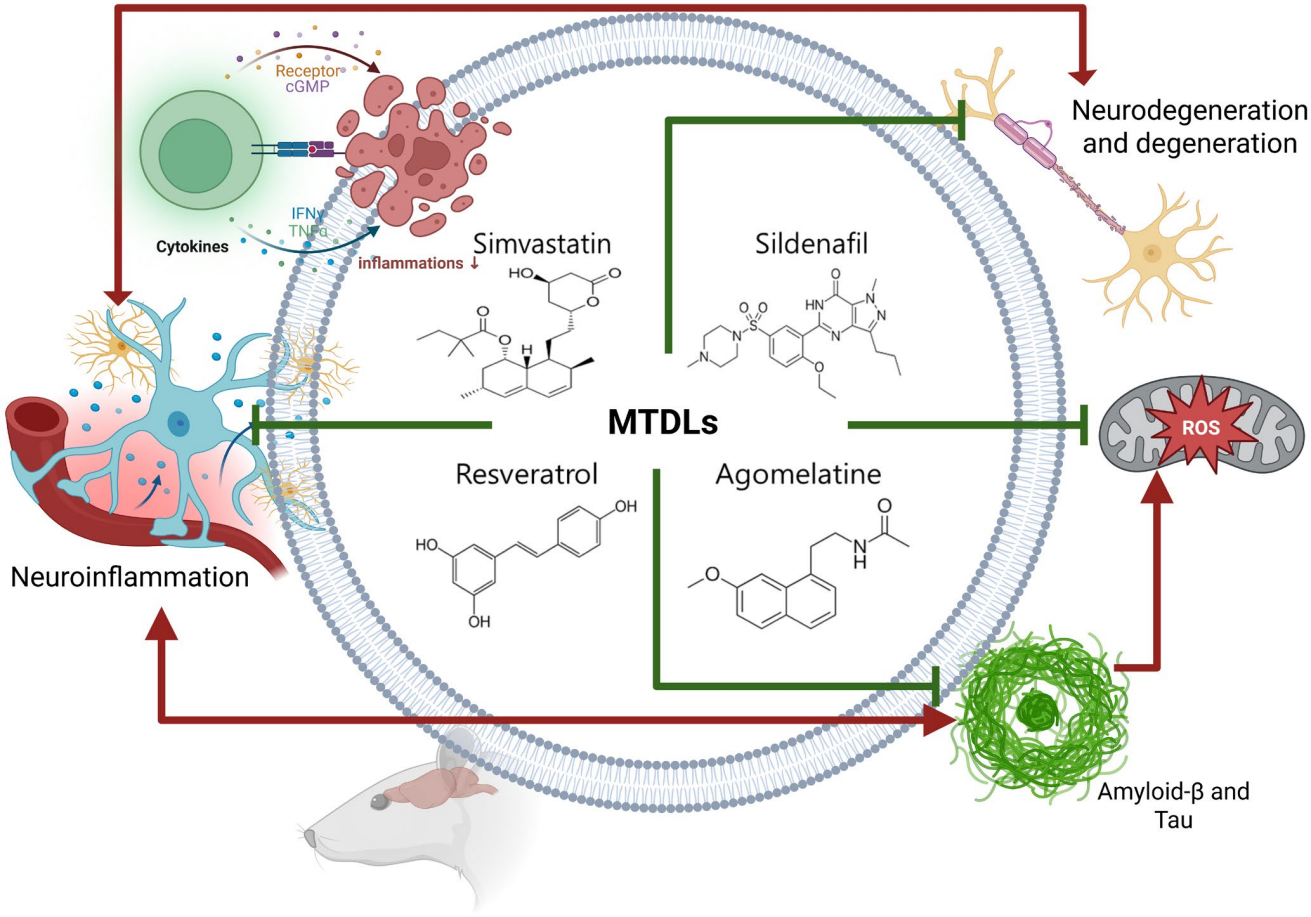
Therapeutic potential of multi‐target agents acting on receptor signaling, oxidative stress, cholesterol metabolism, and pathways in Alzheimer's disease. Agomelatine, Resveratrol, Simvastatin, and Sildenafil exert their effects through modulation of melatonin/serotonin receptors, antioxidant and anti‐amyloid actions, regulation of cholesterol metabolism and inflammation, and enhancement of cGMP signaling, respectively. These mechanisms simultaneously target the major pathological hallmarks of Alzheimer's disease, including amyloid‐β accumulation, tau hyperphosphorylation, and chronic neuroinflammation. Such multi‐target strategies highlight their potential as therapeutic approaches for Alzheimer's disease.

In this review, we summarized the major pathological factors implicated in the onset and progression of Alzheimer's disease, including amyloid‐β deposition, tau hyperphosphorylation, and chronic neuroinflammation. We further discussed the rationale for multi‐target‐directed ligands (MTDLs) as promising therapeutic strategies capable of simultaneously modulating these interconnected pathways. In particular, we highlighted representative agents such as agomelatine, resveratrol, simvastatin, and sildenafil, which exhibit multitarget activities including anti‐amyloid, anti‐tau, antioxidant, and anti‐inflammatory effects (Figure 2).

Alzheimer's disease (AD) has complex pathological mechanisms, and neuroinflammation plays a significant role in disease progression. In addition to Aβ plaque accumulation and abnormal tau protein aggregation, chronic inflammatory responses driven by persistent activation of microglia and astrocytes accelerate neurodegeneration and contribute to cognitive decline. Current research suggests that the pathological characteristics of AD necessitate approaches that simultaneously target multiple pathways, rather than single‐target therapies.

In addition to classical central nervous system–focused mechanisms, emerging evidence highlights the importance of systemic and peripheral factors in Alzheimer's disease pathophysiology. In particular, metabolic regulation, gut–brain interactions, and glial nutrient‐sensing mechanisms have been shown to influence neuroinflammation, amyloid‐β accumulation, and tau pathology [160]. These findings suggest that AD should be understood not solely as a CNS‐restricted disorder but as a multifactorial disease involving dynamic interactions between central and peripheral systems [161]. Incorporating these additional axes further strengthens the rationale for multi‐target therapeutic strategies that can simultaneously modulate both central and systemic contributors to disease progression.

Multi‐targeting ligands (MTDLs) are attracting attention for their potential to simultaneously modulate multiple pathological pathways in Alzheimer's disease. In particular, agents that control neuroinflammation and inhibit Aβ accumulation and tau protein hyperphosphorylation are emerging as important candidates for the treatment of Alzheimer's disease. The multi‐targeting agents mentioned above have demonstrated efficacy in previous studies, and if they overcome the limitations mentioned above, they have the potential to suppress Aβ accumulation, modulate inflammatory responses, and exert neuroprotective effects.

Furthermore, various molecules associated with neuroinflammation modulate the activation of microglia and astroglia, thereby becoming important targets for suppressing neuroinflammation and promoting neuroprotection. The development of MTDLs that simultaneously target these pathways may offer new hope in the treatment of Alzheimer's disease. Melatonin derivatives, such as agomelatine, suppress inflammation through serotonin and melatonin receptors, and PDE5 inhibitors, such as sildenafil, have the potential to slow the progression of Alzheimer's disease by simultaneously improving cerebral blood flow and modulating inflammatory responses.

Multi‐target approaches represent a significant advancement in the treatment of Alzheimer's disease and will likely overcome the limitations of existing single‐target therapies [162]. Future research will require more thorough preclinical and clinical trials of these agents to assess their efficacy and safety and confirm their potential for practical therapeutic application. Furthermore, in the development of MTDLs, assessing the pharmacological properties of the drugs, along with their ability to effectively cross the blood–brain barrier (BBB), will be crucial.

In conclusion, the future of Alzheimer's disease treatment will likely depend, at least in part, on the utilization of multi‐target ligands, alongside other emerging therapeutic strategies. By exploring various MTDLs as novel therapeutic approaches to more effectively address the complexities of AD, exploring the underlying mechanisms of neuroinflammation in AD, highlighting key compounds with multi‐target activity, and building on preclinical and clinical studies, we believe this approach will play a crucial role in improving the quality of life for patients with Alzheimer's disease and reducing the societal burden of age‐related diseases.

## Author Contributions

Jaewoon Jung, Yebeen Kim, Asifiwe Clarisse Cirunduzi, and Seonghyun Yoon wrote the original draft and validated the manuscript. Jaewoon Jung drew all figures. Seokyoung Bang and Seung‐Hoon Yang conceptualized, reviewed, and edited the manuscript.

## Funding

This work was supported by the Basic Science Research Program through the National Research Foundation of Korea (RS‐2026‐25481528 to S‐H.Y., RS‐2024‐00350442 and RS‐2024‐00411952 to S.B.); Korea Environment Industry & Technology Institute (KEITI) through Technology Development Project for Safety Management of Household Chemical Products, funded by Korea Ministry of Environment (MOE) (RS‐2025‐02223058 to S.B.).

## Conflicts of Interest

The authors declare no conflicts of interest.

## Data Availability

The data and figures that support the findings of this study are available from the corresponding author, Seung‐Hoon Yang upon reasonable request.
